# Huangqi Injection (a Traditional Chinese Patent Medicine) for Chronic Heart Failure: A Systematic Review

**DOI:** 10.1371/journal.pone.0019604

**Published:** 2011-05-06

**Authors:** Shufei Fu, Junhua Zhang, Francesca Menniti-Ippolito, Xiumei Gao, Francesca Galeotti, Marco Massari, Limin Hu, Boli Zhang, Rita Ferrelli, Alice Fauci, Fabio Firenzuoli, Hongcai Shang, Ranieri Guerra, Roberto Raschetti

**Affiliations:** 1 Institute of Traditional Chinese Medicine, Evidence-Based Medicine Center and Tianjin State Key Laboratory of Modern Chinese Medicine, Tianjin University of Traditional Chinese Medicine, Tianjin, China; 2 National Centre of Epidemiology, Surveillance and Health Promotion, National Institute of Health, Rome, Italy; 3 External Relations Office, National Institute of Health, Rome, Italy; 4 Centre for Natural Medicine, San Giuseppe Hospital, Empoli, Italy; Universidad Peruana Cayetano Heredia, Peru

## Abstract

**Background:**

Chronic heart failure (CHF) is a global public health problem. Therefore, novel and effective drugs that show few side-effects are needed. Early literature studies indicated that Huangqi injection is one of the most commonly used traditional Chinese patent medicines for CHF in China. As a large number of clinical studies has been carried out and published, it is essential to evaluate the effectiveness and safety of Huangqi injection. Therefore, we carried out this systematic review under the support of the framework of the Joint Sino-Italian Laboratory (JoSIL).

**Objectives:**

To evaluate the efficacy and safety of Huangqi injection for CHF according to the available scientific knowledge.

**Methods:**

An extensive search including PubMed, EMBASE, CBM, the Cochrane Library and Chinese literature databases was performed up to July 2008. Clinical trials regarding Huangqi injection for the treatment of CHF were searched for, irrespective of languages. The quality of each trial was assessed according to the Cochrane Reviewers' Handbook 5.0, and RevMan 5.0 provided by the Cochrane Collaboration and STATA 9.2 were used for data analysis.

**Results:**

After selection of 1,205 articles, 62 RCTs and quasi-RCTs conducted in China and published in Chinese journals were included in the review. The methodological quality of the trials was low. In most trials inclusion and exclusion criteria were not specified. Furthermore, only one study evaluated the outcomes for drug efficacy after an adequate period of time. For these reasons and because of the different baseline characteristics we did not conduct a meta-analysis.

**Conclusions:**

Although available studies are not adequate to draw a conclusion on the efficacy and safety of Huangqi injection (a traditional Chinese patent medicine), we hope that our work could provide useful experience on further studies on Huangqi injections. The overall level of TCM clinical research needs to be improved so that the efficacy of TCM can be evaluated by the international community and possibly some TCM can enter into the international market.

## Introduction

Chronic heart failure (CHF) is the end-stage of various heart diseases that arise for many reasons. The American Heart Association (AHA) has defined CHF as a complex clinical syndrome that can result from any structural or functional cardiac disorder that impairs the ability of the ventricle to fill with or eject blood [1].

A Report from the American Heart Association Statistics Committee and Stroke Statistics Subcommittee indicates that heart failure (HF) incidence approaches 10 per 1,000 of the population over 65 years of age. After HF is diagnosed, survival rates are lower in men than in women, but fewer than 15% of women survive more than 8 to 12 years. The estimated direct and indirect cost of HF in the United States in 2008 is $34.8 billion [2].

The European Society of Cardiology (ESC), representing countries with a population of over 900 million, estimates at least 10 million patients with HF in these countries. The prognosis of HF is uniformly poor if the underlying problem cannot be rectified. Half of the patients carrying a diagnosis of HF will die within 4 years, and more than half of those with severe HF will die within 1 year [3].

In 2000, the United States, China, Australia and Thailand jointly carried out an international cooperation research program on cardiovascular disease in Asia (InterASIA). The adult population sampled was collected from 10 Provinces in China (five in the north, and five in the south). The urban and rural populations accounted for 50% of each, as did the proportion of males to females. The results showed that, on a total of 15,518 adults surveyed (35–74 years old) the prevalence of CHF was 0.9% for the general population, 0.7% for the males, and 1.0% for the females. The risk of CHF was higher in northern than southern China (*P*<0.01) and was higher in urban than rural areas [4].

The AHA, the ESC and other international organizations have actively developed and updated the guidelines for the diagnosis and management of CHF, in order to provide evidence for the diagnosis and treatment of HF [1], [3]. At present, the effectiveness of conventional treatments for CHF has been proven (e.g. angiotensin converting enzyme (ACE) inhibitors, β-blockers and diuretics). Despite several therapeutic approaches in CHF management have led to an important reduction of cardiovascular morbidity and mortality, CHF remains the only cardiovascular disease with an increasing hospitalization burden and an ongoing drain on health care expenditures [5]. Therefore, it remains necessary to seek novel effective drugs.

In some “traditional” medicines plant derived products may have a therapeutic use in heart failure treatment. A systematic review on Hawthorn has reported a significant benefit in symptom control and physiologic outcomes as an adjunctive treatment for CHF [6]. Studies on animal models of human disease suggest that resveratrol has the potential to decrease cardiovascular symptoms in patients with myocardial and cardiomyopathies [7]. Curcumin may protect against the pathological changes occurring with atherosclerosis [8], and the potential therapeutic use of cannabinoids in heart failure has been studied [9].

A previous non-systematic review of the literature to individuate TCM herbs used for treatment of CHF was performed from some of the authors of this review, many Chinese patent medicines and traditional prescriptions (decoctions of Chinese herbs) were listed. Among the others, Huangqi injection was seen to be one of the most commonly used Chinese patent medicines for the treatment of CHF in clinical practice [10] as complementary treatment to recommended Western therapies. So we decided to perform a systematic review on Huangqi injection for CHF.

Huangqi injection is a preparation of an extract of *Radix Astragali*: the major components are astragalosides [11], and the other pharmacological ingredients include polysaccharides, flavones and amino acids. Modern pharmacological research has shown that Huangqi injection can enhance myocardial contractility, improve circulation, protect myocardial cells and regulate immunity [12], [13].

*Astragalus membranaceus* (Fisch) Bge var. Mongolicus (Bge) Hsiao, is a typical Traditional Chinese Medicine plant, used as food, and present since many years on the Western market (in Europe and USA) as food supplement. *Astragalus membranaceus* has being used for thousands of years in China and East Asia also for kidney diseases, and in modern Chinese medicine, it seems to have renal protective effect in diabetic nephropathy [14].

The extract of the Astragalus root is usually used also in Western phytotherapy as galenic preparations, containing dried extract standardized in polysaccharides, the substances that are mostly considered responsible for the presumed immunostimulant properties [15]: it is in particular used for recurrent respiratory diseases or as therapeutic complement in cancer treatment [16].

To date, a large number of clinical studies have been reported in the literature. We have here carried out a systematic review to evaluate the effectiveness and safety of Huangqi injection for CHF in a joint Sino-Italian collaboration.

Traditional Chinese Medicine (TCM) has been used for many centuries, and it is still widely used today in countries of south and east Asia for the treatment of people with CHF.

With the purpose of investigating the appropriate scientific evidence for some specific TCM the Italian National Institute of Health and the Tianjin University of Traditional Chinese Medicine have implemented a common project, the Joint Sino-Italian Laboratory (JoSIL) on TCM. This systematic review was conducted within the framework of JoSIL.

## Methods

### Types of studies

Randomized and quasi randomized clinical trials (RCTs) to evaluate Huangqi injection for CHF up to July 2008 were searched, irrespective of languages. With the term “quasi-RCT” we intend trials that use methods to allocate patients such as alternation, case record numbers, dates of birth, etc. For these trials there is a greater risk of selection bias compared with RCTs.

### Types of participants

Trials including adult patients with CHF were eligible, irrespective of the etiology. CHF had to be diagnosed according to the ACC/AHA 2005 Guidelines for the Diagnosis and Management of CHF, or according to the corresponding diagnostic criteria in China [1], [17]. Trials that included patients with acute-HF were excluded.

### Types of interventions

We included in our review only trials that tested Huangqi injection plus conventional treatments *versus* conventional treatments alone for CHF. The studies that used hospital preparations of Huangqi injection were excluded. Trials in which the type of preparation was not specified (either from a pharmaceutical company or hospital) were included.

### Study selection

To identify all of the relevant studies, an extensive search was performed in July 2008. We searched the Cochrane Central Register of Controlled Trials (2008, issue 3), PubMed (1950–2008), EMBASE (1985–2008) and the Chinese Biomedical Database (1978–2008). We also searched other Chinese journal databases (Chinese Journal Full-Text Database (1979–2008), Chinese Science and Technology Journal Database (1989–2008) and Wanfang Data Info Site) to identify theses and conference articles. To reduce omissions, we made an extensive electronic database search, followed by handsearch. The keywords were “Huangqi OR *mongolian milkvetch root* OR *Astragalus membranaceus* OR astragalus extract OR *Radix Astragali*” AND “chronic heart failure, cardiac dysfunction, cardiac inadequacy, cardiac insufficiency, heart insufficiency, ventricular dysfunction, cardiac failure, myocardial failure, congestive heart failure, heart decompensation”. These terms were used as free-text terms (translated into Chinese) to search some of the Chinese databases. Free-text and MeSH terms were used to search PubMed and Cochrane Library. Free-text and Emtree terms were used to search EMBASE. We also checked the references of published studies to identify additional trials.

Two reviewers (SF, JZ) independently examined the titles and abstracts of the trials for inclusion, based on the selection criteria outlined above. The full texts of articles were retrieved if there was any doubt whether an article should be included or not. Inconsistencies were solved through discussion. The selected trials that were claimed to be randomized were retrieved and then examined for confirmation that they were indeed correctly randomized. A table with the English translation of all the titles and the English abstract was reviewed by other two investigators (MM, FMI).

### Quality assessment of trials included

A quality assessment was carried out for all the retrieved studies. Quality in a systematic review essentially refers to the absence of biases.

The main biases in a clinical trial can derive from systematic differences between comparison groups in: measured or unmeasured baseline characteristics because of the way participants were selected or assigned (selection bias); care provided apart from the intervention being evaluated (performance bias); how outcomes are ascertained, diagnosed or verified (detection bias); withdrawals or exclusions of participants from the results of a study (attrition bias).

To assess the methodological validity of the studies included in this review the following aspects were evaluated (according to a binary score presence/absence): randomization, allocation concealment, blinding and description of follow-up. According to Cochrane reviewers' Handbook [18] three categories were defined: A-all quality criteria met: (low risk of bias); B-one or more of the quality criteria only partly met (moderate risk of bias); C-one or more criteria not met (high risk of bias).

Articles were assessed by two reviewers (JZ and SF) independently. Disagreements were resolved by consultation with a third reviewer (XG).

### Data extraction

The information on patients, methods, interventions, outcomes and results was extracted and summarized by two reviewers (SF and JZ) independently using a standardized data extraction form. Disagreements were resolved through discussion among all Chinese investigators. For dichotomous outcomes, the number of responders and the total number of participants for each study arm were extracted. For continuous outcomes (e.g. left ventricular ejection fraction), the mean change and standard deviation for the mean in each group of the trial were extracted along with the total number.

### Data analysis

In this study, statistical analysis was performed using software provided by the Cochrane Collaboration (Review Manager 5.0) [19] and STATA 9.2 software [20].

For measurement data, we took the differences before and after treatment for statistical analysis. Dichotomous data are presented as rate ratio, while continuous data are expressed as mean difference (MD), all with 95% confidence intervals (CI). A funnel plot was used to investigate potential publication bias.

## Results

### Search flow

According to the search strategy we identified 1,205 potentially relevant studies. By reading titles and abstracts, we excluded 728 studies that were clearly duplicates, review articles, case reports or non-clinical studies.

**Figure 1:**
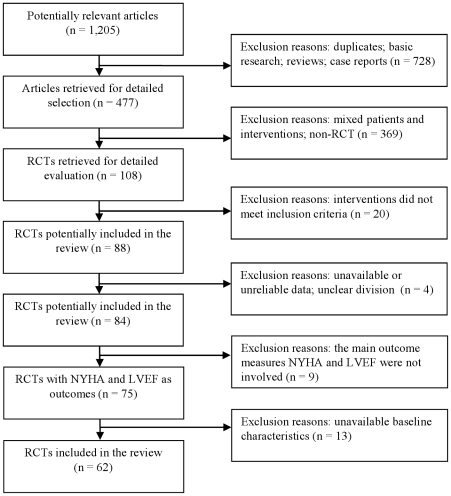
Flow diagram of the article selection for this study.

After the full text reading, 369 articles were excluded because non-RCT or patients not satisfying the inclusion criteria. Twenty were excluded because the intervention did not meet the inclusion criteria and 4 because of not reliable data. The improvement of the New York Heart Association (NYHA) classification or the Left Ventricular Ejection Fraction (LVEF) were considered as outcomes in seventy five (89%) of the remaining 84 trials. For these reasons only trials reporting these outcomes were analyzed. Thirteen trials were excluded because the baseline data for outcome measures (NYHA classification and LVEF) was not available (Figure 1).

The baseline characteristics (e.g. sex, age, course of disease) of the patients included in the trials reporting NYHA classification and LVEF were described only in 62 trials. The following results are only related to these trials (Table S1) [21]–[82].

We did not consider other outcomes such as cardiac output, speed of ventricular diastolic filling, left ventricular volume, heart rate, systolic and diastolic blood pressure or other cardiovascular endpoint because less frequently listed in the trials and not so well described. No trial had any evaluation of Quality of Life. All of the included studies were carried out in China over the past 14 years (1995–2008). Sixteen of these studies had an English abstract and two of them were conference articles [39], [75]. All of these studies indicated randomization, but most of them were quasi-RCTs with a single-center, parallel-design.

### Characteristics of the trials included

The number of participants included in each of the studies ranged from 30 to 140 (only one study included 532 patients), with a total of 5,548 patients (2,950 patients in the investigational groups) in the 62 studies. The mean proportion of males present in the trials was 63.3%. The age of the participants ranged from 18 years to 89 years, with a mean age of 60.9 years. Fifty-eight studies mentioned the etiology (12 studies only included patients with pulmonary HF). Twenty-one studies reported the duration of the disease: from one month to 28 years.

In seven of the trials, the inclusion criteria were specified, and in three the exclusion criteria were defined. Only four of the trials reported a description of all the subjects selection criteria (e.g. diagnostic, inclusion criteria, exclusion criteria). The criteria of termination and completion were not mentioned in any of the studies.

Five studies, among the 62, reported mortality data.

All patients included in the trials were not in an acute exacerbation of CHF, and they were hospitalized to prevent acute exacerbation of CHF. During these non-acute admissions the trials were conducted.

The studies included interventions with Huangqi injection plus routine treatments *versus* routine treatments alone. Routine treatments referred to treatment according to the guidelines for CHF, including cardiotonics, diuretics, Angiotensin II receptor blockers (ARBs), (ACE) inhibitors and β-blockers [1], [3]. Thirty six studies mentioned the brand of the Huangqi injections, which were from five pharmaceutical companies. Nine studies clarified the lot number of the Huangqi injection and 23 studies described the doses conversion relationship between the injection and the medical herbs. The doses of Huangqi injection used ranged from 10 ml to 60 ml. The duration of treatment ranged from 7 days to 30 days, with a median treatment duration of 14 days. A variety of outcome measures were reported. The evaluation of the outcomes was performed at the end of the treatment. Only in 2 trials the follow up period was mentioned (from 3 to 6 months).

### Methodological quality

All of the studies included indicated randomization, with three of the trials reporting that the random sequence was generated by a random digits table, and four by the patient treatment order. No trial described allocation concealment. Two of the trials mentioned that they were single blind, but included no descriptions of the procedures. Two of the trials described withdrawals with no intention-to-treat analyses. None of the trials described any methods of assessing compliance.

The analysis was only focused on trials that considered as outcome measure an improvement of at least one class on the NYHA classification or on the LVEF. These outcomes were chosen because they were considered the most relevant for a clinical point of view in the evaluation of the effectiveness of drugs.

In general, the methodological quality of the included trials was poor. In most trials inclusion and exclusion criteria were not specified. Furthermore, only one study evaluated the outcomes for drug efficacy after an adequate period of time. There was not any study of sufficient quality that could be highlighted.

For these reasons and because of the different baseline characteristics we did not conduct a meta-analysis.

### Effectiveness

The “effective rate” (ER) was the most commonly used measure to evaluate efficacy. In 49 studies where this measure was reported an “effective rate ratio” (ERR) was calculated as the ratio between the proportion of responders in the treatment group and the proportion of responders in the control group. The responders were defined as an improvement of at least one class on the NYHA classification. In 35 trials responders were defined as improvements of NYHA classification and some TCM symptoms (such as coated tongue, color of lips, cough and edema). Since ER reported in the trials considering NYHA classification with or without some TCM symptoms were similar we did not present separate figures for the two groups of studies. The other outcome measure was the LVEF. The mean difference (MD) of LVEF before and after the study treatment was measured in 23 studies. The effect measure was estimated as the mean difference in the intervention and control groups. In 10 studies both outcomes were considered.

### NYHA classification

**Figure 2:**
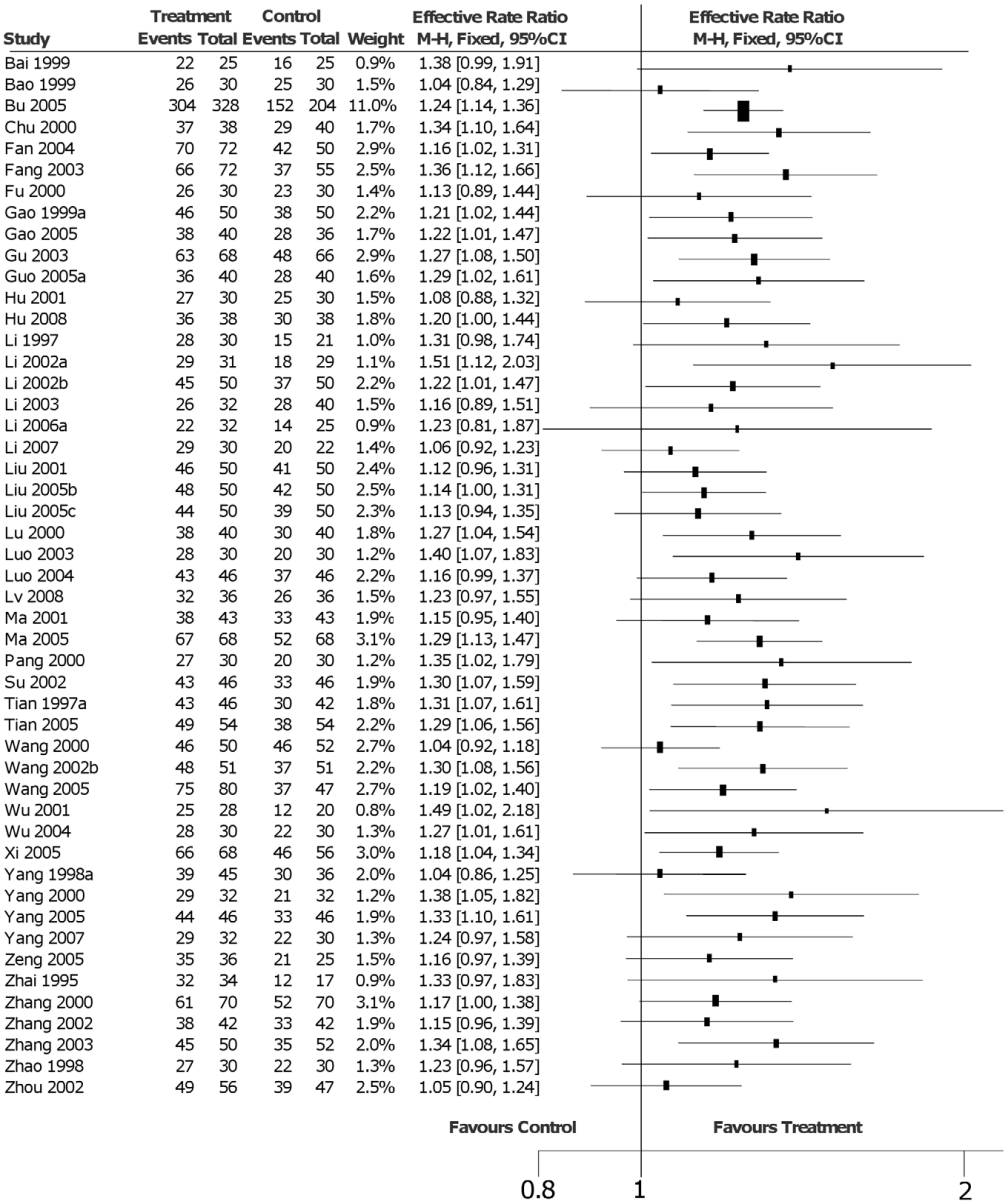
Effective rate analysis based on improvements of classification in NYHA.

As illustrated in figure 2, forty-nine trials with 4,584 patients (2,435 patients in the active treatment group) selected as outcome measure an improvement of at least one class on the NYHA classification. For this indicator 25 studies were statistically significant, showing that Huangqi injection plus routine treatment can improve cardiac function and clinical symptoms. There was not statistically significance in the other 24 studies.

### LVEF

**Figure 3:**
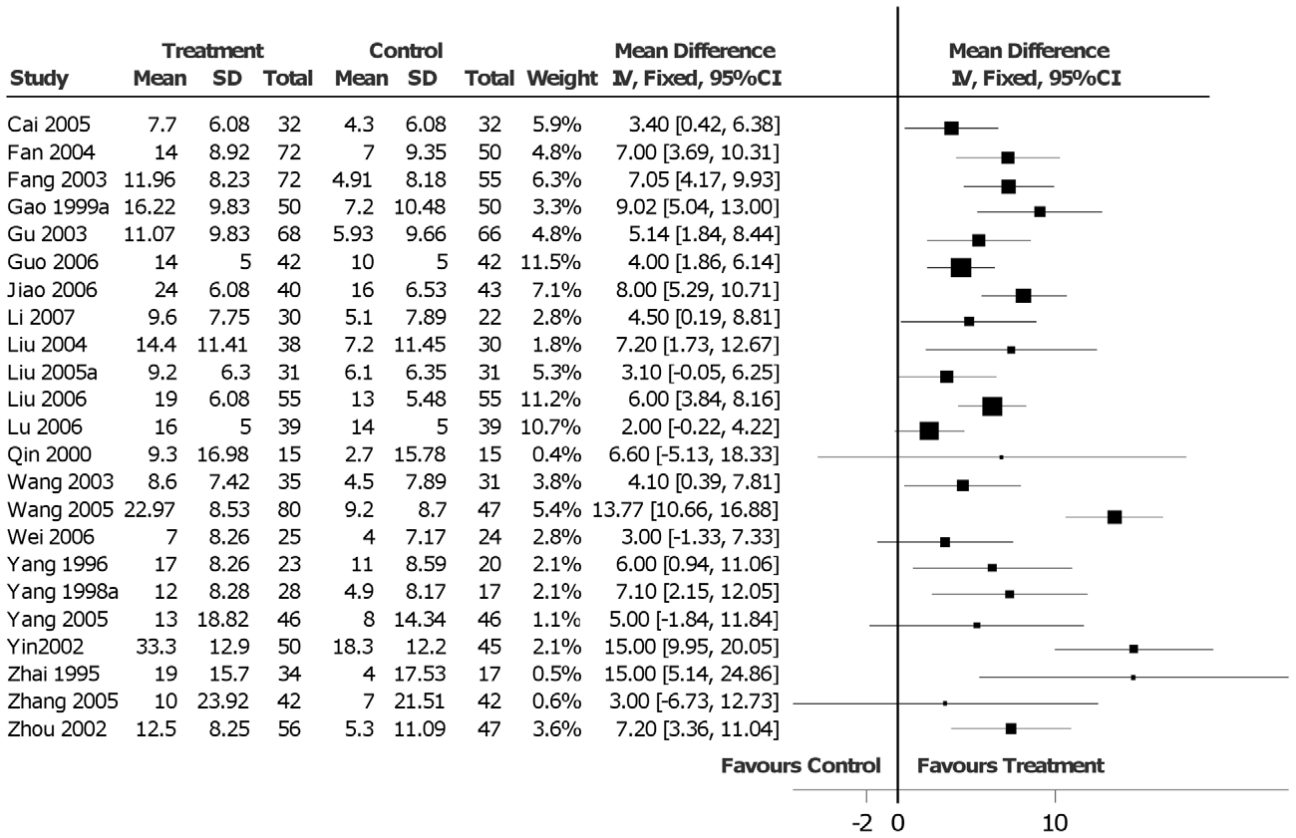
Data analysis of left ventricular ejection fraction.

LVEF is the ratio of the stroke volume and the left ventricular end-diastolic volume. It is frequently used for the assessment of HF and for the evaluation of drug efficacy. Twenty-three studies reported the outcomes for LVEF. Seventeen studies showed that Huangqi injection combined with routine treatment was better than routine treatment used alone for the increase in LVEF, while six studies were not statistical significant (Figure 3).

### Other outcomes

Five [55], [62], [76], [77], [82] of the 62 studies reported the mortality data. Though mortality is a very important indicator, these studies just reported the number of deaths rather than the causes or only reported the causes very imprecisely. Furthermore, the studies were not designed for the evaluation of mortality as primary outcome.

### Safety analysis

Adverse events were not mentioned in 30 of the studies (48.4%). Twenty-eight studies (45.2%) reported that no adverse reactions were seen at the end of treatment. In four studies, various adverse reactions were seen for 15 patients in the control group and 11 patients in the test group.

The side-effects in the routine treatment group included dry cough (4/47), bradycardia (2/47), low blood pressure (2/47) and digitalis poisoning (6/36). The main side effects related to Huangqi injection were mainly discomfort of the gastrointestinal tract. Three studies [35], [64] reported dizziness, and facial flushing as side effects, with a rate of 5.9% (7/118). Three patients reported mild nausea in two studies [64], [74], with an incidence rate of 3.8% (3/80). After treatment or slowing down of the intravenous drip, the patients recovered.

### Funnel plot

**Figure 4:**
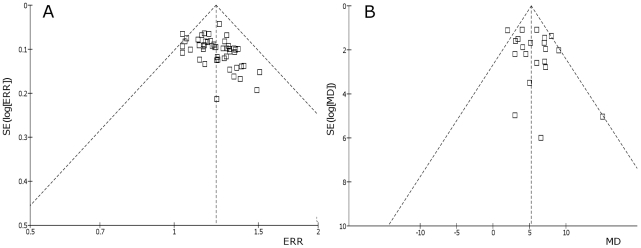
Funnel plots of ERR (A) and LVEF Mean Difference (B).

Funnel plots based on the data for the ERRs and MD of LVEF were elaborated in Figure 4. The figures are asymmetrical indicating that potential publication bias might influence the results of this review.

## Discussion

Limitation of our review was the underlying concern about the quality of the included studies.

In our systematic review, in fact, it was not possible to find well-designed trials to evaluate the efficacy of Huangqi injection for CHF, and, thus, meta-analysis was not performed.

All the trials use an A+B versus B design where patients are randomized to receive a control treatment (the Control Group) versus a control treatment plus the experimental treatment (the Treatment Group). This kind of design is likely to generate false positive results [83].

Formal inclusion/exclusion criteria were reported only in 22.6% of the studies. Baseline information on disease duration was available only in 21 trials. None of the trials included provided information on clinical stability of the patients recruited in the weeks preceding trial recruitment. Furthermore, no information on persistence on previous treatments is available in any of the studies included and it can not be excluded that in some of the participants routine treatment began concomitantly with Haungqi treatment. The lack of baseline information may lead to selection bias and not comparable baseline.

In general we considered not reasonable in our review to calculate a pooled average result across studies because of their high clinical heterogeneity (i.e. dissimilarity in disease severity, previous episodes of medical care, co-morbidities, co-interventions, etc).

Another common shortcoming was the inadequate description of the randomisation procedures. Although all trials claimed to have performed randomization, only three trials reported the method to generate the allocation sequence (random digits table) and the remaining trials did not give any details of the randomization method. Blinding was mentioned in two trials without describing the methods of allocation concealment. Thus, whether the randomization was effectively conducted in these trials was doubtful. Inappropriate randomization or allocation concealment can lead to selection bias. Not controlling for blinding could deal with an overestimation of the effect of the experimental treatment, unless the assessment of the outcome is performed by a researcher not involved in the treatments allocation. All trials were conducted only in one clinical centre. Dropout cases were reported in three trials and were not mentioned in the others. It was not clear whether these trials had any dropout cases or whether they were simply not reported. Having dropout cases without reporting can result in attrition bias. Only 51.6% of the studies described the occurrence of adverse events.

As far as the quality of Huangqi preparations are concerned, even though the studies that adopted hospital preparations were excluded from our review, 26 of the included studies did not mention the source of the Huangqi injection. Quality control of herbal preparations is crucial for the critical appraisal of the results in this kind of studies. In order to assess the efficacy of a specific product in a clinical study, all participants should be given exactly the same intervention in terms of product identity, purity, dosage and formulation.

From a clinical standpoint, only 5 of the studies included in our revision approached important end-points such as mortality, but the causes were not clearly reported. All the trials were actually focussed on ancillary or surrogate outcomes. The evaluation of clinical symptoms through the classification system of the New York Heart Association is subject to placebo effect and moreover most of the included studies were not blinded. Although ventricular dysfunction is the hallmark of low output cardiac failure, hemodynamic data, such as ejection fraction, should be regarded as supportive only. Changes in various invasive and non-invasive measures of ventricular performance have not shown to correlate closely with each other and many of them do not correlate with clinical symptoms or functional capacity [84]; for these reasons the value of these measures in evaluating the efficacy of drugs in patients with cardiac failure during long-term treatment is very limited [84].

In all the trials the duration of therapy and follow-up was indeed too short to allow to achieve conclusive results. Only in 2 trials included in our review there was a follow-up period (range 3–6 months) duration, while in the other studies the outcomes were evaluated only at the end of the treatment (range 7–30 days). In order to evaluate drug efficacy for CHF, long-term improvement (at least 6 months) of CHF-specific clinical symptoms is needed [84]. Some drug classes have shown to increase mortality in the long-term application despite a short term improvement in clinical symptoms [84].

Adverse events were reported only in 50% of trials, indicating an incomplete evaluation of the safety profile of Huangqi injection, as well as a poor quality in the reporting.

Overall, because of the poor methodological quality of the studies, the results of the trials included in this systematic review are likely to be influenced by many biases. Furthermore, since the review included only articles published on the Chinese literature and no primary articles reporting negative results were found, a location bias cannot be excluded (i.e. trials published in low or non-impact factor journals are more likely to report significant results than those published in high-impact mainstream medical journals).

Some research on TCM could move to an efficacy-driven approach. Randomized, controlled trials, when appropriately designed, conducted, and reported, represent the gold standard in evaluating health care interventions.

Well-designed and properly conducted RCT provides high-quality “raw materials” for conducting systematic reviews and health technology assessment. Poorly designed and reported trials usually exaggerate the treatment effects which will mislead clinical decision making.

In 2008, Xu et al assessed the randomized clinical trials published in the five leading Chinese medical journals indexed by MEDLINE and found that only 22 articles (15.5%) reached a high quality grade (> = 3 points) according to the Jadad scale [85].

Randomized clinical trials in TCM area often show methodological weaknesses: absence of sample size estimation, failure in use (or reporting) randomization as well as unclear study objective and hypothesis are common [86], [87].

Because of the low methodological quality of trials and selective publication of positive results, the efficacy of most traditional therapies remains at the best uncertain.

Journals in China should obey international criteria for publication (such as CONSORT, STROBE, PRISMA, etc) and require documentation of ethics approval and clinical trial registration before manuscript acceptance.

Ethical issues are increasingly gaining attention from Chinese researchers. However, in the studies included in our SR ethical issues were not mentioned. Only one study [65] mentioned that the informed consent was obtained.

Chinese Government has made research into traditional medicine a priority area and, with funding increasing by 20% a year, China now has more investment in research and development than any other country except the USA [88], [89]. Chinese regulations and guidelines do not substantially differ from those in Europe or the USA but their implementation in daily research and clinical practice do not seem to be comparable, at the moment, with international standards.

International collaboration should be encouraged, promoted and financed from the governments in order to improve research.

## Supporting Information

Table S1 **Characteristics of the studies included.**
(DOC)
