# Supplementary material for: Huangqi Injection (a Traditional Chinese Patent Medicine) for Chronic Heart Failure: A Systematic Review
Source: PLoS One. 2011 May 6;6(5):e19604. doi: 10.1371/journal.pone.0019604 (PMC3089614; doi:10.1371/journal.pone.0019604)
Supplement: Table S1 — Characteristics of the studies included. (DOC) [file pone.0019604.s001.doc]

**Table S1. Characteristics of the studies included**

| **ID** | **Sample Size** | | **Males (%)** | | **Age: Mean (Range)** | | **Disease Duration: Mean (Range)** | | | **Treatment(*)** | | **Outcomes** |
| --- | --- | --- | --- | --- | --- | --- | --- | --- | --- | --- | --- | --- |
| **Author Year** | **T** | **C** | **T** | **C** | **T** | **C** | **T** | **C** | | **HQ ml iv gtt die**  **+ RT** | **Duration** |  |
| Bai 1999 ^21^ | 25 | 25 | 48 | 56 | 69.9 (27 - 82) | 61.7 (25 - 78) |  |  | | 10-20 | 10-14d | NYHA |
| Bao 1999 ^22^ | 30 | 30 | 93 | 87 | 71.6 (60 - 89) | 70.8 (60 - 70) | 8.8y (1y - 22y) | 7.9y (2y - 20y) | | 10 | 14d | NYHA |
| Bu 2005 ^23^ | 328 | 204 | 60 | 63 | 68.6 (60 - 89) | 66.9 (61 - 86) | 3.4y (2y - 11y) | 4.1y (1y - 12y) | | 20-40 | 14d | NYHA |
| Cai 2005 ^24^ | 32 | 32 | 63 | 56 | 64.2 | 63.5 |  |  | | 40 | 4w | LVEF |
| Chu 2000 ^25^ | 38 | 40 |  |  | (48-81) | |  |  | | 30-40 | 14d | NYHA |
| Fan 2004 ^26^ | 72 | 50 | 82 | 82 | 67.5 | 65.8 |  |  | | 20 | 14d | NYHA; LVEF |
| Fang 2003 ^27^ | 72 | 55 | 67 | | 65.7 (36 - 78) | |  |  | | 40 | 15d | NYHA; LVEF |
| Fu 2000 ^28^ | 30 | 30 | 53 | 57 | 52 (31 - 73) | 52 (32 - 72) |  |  | | 20 | 14d | NYHA |
| Gao 1999a ^29^ | 50 | 50 | 62 | | 56.2 (30 - 76) | |  |  | | 20 | 15d | NYHA; LVEF |
| Gao 2005 ^30^ | 40 | 36 | 51 | | 70 (49 - 82) | | (0.5y - 10y) | | | 40 | 30d | NYHA |
| Gu 2003 ^31^ | 68 | 66 | 52 | | 55.8 (42 - 76) | | (8m - 10y) | | | 40 | 4w | NYHA; LVEF |
| Guo 2005a ^32^ | 40 | 40 | 70 | 80 | 60.3 (52 - 76) | 59.3 |  | |  | 20 | 7-10d | NYHA |
| Guo 2006 ^33^ | 42 | 42 |  |  | 52.3 (40 - 74) | |  | |  | 30 | 10d | LVEF |
| Hu 2001 ^34^ | 30 | 30 | 80 | | (52-80) | | (3y - 28y) | | | 40 | 14d | NYHA |
| Hu 2008 ^35^ | 38 | 38 | 74 | 76 | (54 - 86) | (51 - 82) | (3y - 20y) | | (2y - 19y) | 30 | 14d | NYHA |
| Jiao 2006 ^36^ | 40 | 43 | 53 | 44 | 63.8 (54-87) | 59.9 (49 - 77) |  | |  | 40 | 30d | LVEF |
| Li 1997 ^37^ | 30 | 21 | 77 | 76 | 64.2 (53 - 73) | 66.3 (57 - 74) |  | |  | 20 | 10d | NYHA |
| Li 2002a ^38^ | 31 | 29 | 55 | 55 | 62 (45 - 80) | 67 (48 - 79) |  | |  | 20 | 14d | NYHA |
| Li 2002b ^39^ | 50 | 50 | 58 | 54 | 64 | 65 | 5.6y | | 5.2y | 20 | 14d | NYHA |
| Li 2003 ^40^ | 80 | 40 | 65 | 70 | 66.7 (28 - 86) | |  | |  | 20 - 60 | 14d | NYHA |
| Li 2006a ^41^ | 32 | 25 | 63 | 56 | 55.2 (40 - 76) | 53.6 (42 - 74) |  | |  | 50 | 14d | NYHA |
| Li 2007 ^42^ | 30 | 22 | 57 | 59 | 56.2 | 55.8 |  | |  | 30 | 14d | NYHA; LVEF |
| Liu 2001 ^43^ | 50 | 50 | 60 | 56 | 45 (32-80) | 46 (30 - 81) | 4.1y (2.5y - 6y) | | 4y (2.3y - 6.1y) | 50 | 14d | NYHA |
| Liu 2004 ^44^ | 38 | 30 | 66 | | 67.3 | |  | |  | 40 | 3w | LVEF |
| Liu 2005a ^45^ | 31 | 31 | 66 | | 61.0 | | 4.5y | | | 40 | 14d | LVEF |
| Liu 2005b ^46^ | 50 | 50 | 72 | 70 | 73.8 (62 - 85) | 72.2 (60 - 84) |  | |  | 40 | 15d | NYHA |
| Liu 2005c ^47^ | 50 | 50 | 60 | | 56.2 (27 - 87) | |  | |  | 50 | 10d | NYHA |
| Liu 2006 ^48^ | 55 | 55 | 56 | | 48.5 (42 - 75) | |  | |  | 30 | 10d | LVEF |
| Lu 2000 ^49^ | 40 | 40 | 55 | 60 | 62 (30 - 75) | 60 (32 - 74) |  | |  | 20 | 10-15d | NYHA |
| Lu 2006 ^50^ | 39 | 39 | 54 | |  |  |  | |  | 40 | 14d | LVEF |
| Luo 2003 ^51^ | 30 | 30 | 60 | 57 | 52 (28 - 76) | 52 (29 - 75) | 5y (2y - 16y) | | 5y (2y - 18y) | 20 - 60 | 14d | NYHA |
| Luo 2004 ^52^ | 46 | 46 | 57 | 48 | 68 (60 - 76) | 71 (63 - 79) |  | |  | 30 | 20d | NYHA |
| Lv 2008 ^53^ | 36 | 36 | 56 | 64 | 61.3 (48 - 73) | 60.8 (45 - 76) |  | |  | 30 | 14d | NYHA |
| Ma 2001 ^54^ | 43 | 43 | 84 | 72 | (53 - 78) | (51 - 83) |  | |  | 50 | 10d | NYHA |
| Ma 2005 ^55^ | 68 | 68 | 65 | 59 | (60 - 76) | (58 - 78) |  | |  | 50 | 7d | NYHA |
| Pang 2000 ^56^ | 30 | 30 | 67 | 67 | 61 (48 - 70) | 60 (49 - 70) | 4.8y | | 4.6y | 20 | 10d | NYHA |
| Qin 2000 ^57^ | 15 | 15 | 60 | 67 | 57 (45 - 71) | 56 (42 - 72) |  | |  | 40 - 60 | 30d | LVEF |
| Su 2002 ^58^ | 46 | 46 | 57 | | (47 - 82) | |  | |  | 30 | 14d | NYHA |
| Tian 1997a ^59^ | 46 | 42 | 89 | 90 | 58.4 (47 - 78) | 57.6 (48 - 77) |  | |  | 20 | 3w | NYHA |
| Tian 2005 ^60^ | 54 | 54 | 87 | 91 | 58.3 (46 - 80) | 57.6 (48 - 77) |  | |  | 20 | 14d | NYHA |
| Wang 2000 ^61^ | 50 | 52 | 57 | | 61.5 (50 - 72) | | 11.5y | | | 20 | 10-15d | NYHA |
| Wang 2002b^62^ | 51 | 51 | 67 | 63 |  |  |  | |  | 40 | 10-14d | NYHA |
| Wang 2003 ^63^ | 35 | 31 | 74 | 77 | 60.9 | 60.6 |  | |  | 40 | 14d | NYHA; LVEF |
| Wang 2005 ^64^ | 80 | 47 | 58 | 53 | 62.5 (40 - 75) | 62.8 (41 - 729 | 4.7y (1y - 10y) | | 4.7y (1y - 9y) | 20 | 4w | LVEF |
| Wei 2006 ^65^ | 25 | 24 | 63 | | 56 (40 - 72) | |  | |  | 40 | 15d | LVEF |
| Wu 2001 ^66^ | 28 | 20 | 42 | | 53.4 (24 - 72) | | 3.2y (0.5y - 10y) | | | 20 - 30 | 7-10d | NYHA |
| Wu 2004 ^67^ | 30 | 30 | 67 | 63 | 62 | 60 | 15.0y | | 14.5y | 30 | 10d | NYHA |
| Xi 2005 ^68^ | 68 | 56 | 57 | 52 | (18 - 83) | (34 - 86) | (6m - 3y) | | (4m - 3.5y) | 30 | 10-15d | NYHA |
| Yang 1996 ^69^ | 23 | 20 | 57 | 65 | 57 (36 - 70) | 53 (32 - 73) |  | |  | 16 | 10d | LVEF |
| Yang 1998a ^70^ | 45 | 36 |  |  | (36 - 78) | |  | |  | 20 | 14d | NYHA; LVEF |
| Yang 2000 ^71^ | 32 | 32 | 63 | 56 | 49 (27 - 75) | 50 (24 - 76) |  | |  | 20 - 50 | 14d | NYHA |
| Yang 2005 ^72^ | 46 | 46 | 59 | 57 | 63 (50 - 73) | 62 (50 - 72) |  | |  | 30 | 10d | NYHA; LVEF |
| Yang 2007 ^73^ | 32 | 30 | 53 | 60 | 62 (49 - 73) | 63 (50 - 72) | 8y (3y - 13y) | | 9y (4y - 15y) | 20 | 3w | NYHA |
| Yin 2002 ^74^ | 50 | 45 | 60 | 56 | (50 - 75) | (47 - 73) |  | |  | 20 | 15d | LVEF |
| Zeng 2005 ^75^ | 36 | 25 | 81 | 80 | 67.5 | 65.8 |  | |  | 40 - 60 | 14d | NYHA |
| Zhai 1995 ^76^ | 34 | 17 | 68 | 71 | 58 (38 - 80) | 57 (41 - 79) | (6m - 3y) | | (6m - 3y) | 20 | 10d | NYHA; LVEF |
| Zhang 2000 ^77^ | 70 | 70 | 61 | 56 | 62.5 (38 - 78) | 61.7 (40 - 80) |  | |  | 20 | 14d | NYHA |
| Zhang 2002 ^78^ | 42 | 42 | 67 | 69 | 50.1 (38 - 82) | 49.3 (36 - 80) | 8.5y (5y - 10y) | | 8.2y (4y - 10y) | 40 | 14d | NYHA |
| Zhang 2003 ^79^ | 50 | 52 | 70 | 69 | 71.5 | 69.5 |  | |  | 60 | 14d | NYHA |
| Zhang 2005 ^80^ | 42 | 42 | 55 | 57 | 61.1 (37 - 75) | 60.3 (36 -78) |  | |  | 30 | 4w | LVEF |
| Zhao 1998 ^81^ | 30 | 30 | 67 | 60 |  |  | 5.1y | | 4.8y | 20 | 14d | NYHA |
| Zhou 2002 ^82^ | 56 | 47 | 66 | 74 | 61.3 (56 - 75) | 59.4 (54 - 73) | (1m -11y) | | (2m - 9y) | 60 | 3w | NYHA; LVEF |

(*) Treatment groups were treated with HQ ml iv gtt die + routine treatment. Control groups were treated with routine treatments. Routine treatment was not speciefied.

**Note:** HQ=Huangqi injection; RT=routine treatment (cardiotonics, diuretics, angiotensin II receptor blockers (ARBs), angiotensin-converting enzyme (ACE) inhibitors and β-blockers); NYHA: New York Heart Association; LVEF: left ventricular ejection fraction.
